# Implementing a novel capture and ligation probe-PCR method in mass screen and treatment to support malaria elimination efforts in the China-Myanmar border region

**DOI:** 10.1186/s12936-023-04449-x

**Published:** 2023-01-19

**Authors:** Xiao-dong Sun, Ya-ling Zhao, Zu-rui Lin, Ye Zhao, Yao-wu Zhou, Shi-gang Li, Xiang-rui Guo, Peng Tian, Kai-xia Duan, Chun-li Ding, Qi-yan Chen, Yuan Sui, Shen-ning Lu, Chris Cotter, Duo-quan Wang, Zhi Zheng

**Affiliations:** 1 Yunnan Institute of Parasitic Diseases, Yunnan Provincial Collaborative Innovation Centre for Public Health and Disease Prevention and Control, Yunnan Provincial Key Laboratory of Vector-borne Diseases Control and Research, Yunnan Provincial Centre of Malaria Research, Puer, 665000 China; 2 Institute of Basic Medical Sciences, Chinese Academy of Medical Sciences; School of Basic Medicine, Peking Union Medical College, Beijing, 100005 China; 3 Yingjiang Centre for Disease Control and Prevention, Yingjiang, 679300 China; 4 Brown School, Washington University, St. Louis, MO USA; 5 WHO Collaborating Centre for Tropical Diseases, National Center for International Research on Tropical Diseases, Key Laboratory of Parasite and Vector Biology, Ministry of Science and Technology, Ministry of Health, National Institute of Parasitic Diseases, Chinese Center for Disease Control and Prevention, Chinese Center for Tropical Diseases Research, Shanghai, 200025 China; 6 Malaria Elimination Initiative, Institute for Global Health Sciences, University of California, San Francisco, CA USA; 7 Department of Women’s and Children’s Health, Uppsala University, Uppsala, Sweden

**Keywords:** Malaria, Mass screening and treatment, Capture and ligation probe-PCR, Submicroscopic, Field application

## Abstract

**Background:**

Mass screening and treatment (MSAT) for malaria elimination lacks an ideal diagnostic tool to allow sensitive and affordable test of the target population in the field. This study evaluated whether Capture and Ligation Probe-PCR (CLIP-PCR) could be used in a field MSAT in Laiza City, Myanmar.

**Methods:**

On day 0, two dried blood spots were collected from each participant. On day 1, all samples were screened for *Plasmodium* in a 20 m^2^ laboratory with workbench, a biosafety cabinet, a refrigerator, a benchtop shaking incubator and a qPCR machine, by four technicians using CLIP-PCR with sample pooling, at a health clinic of the Chinese bordering town of Nabang. On day 2, all positives were followed up and treated.

**Results:**

Of 15,038 persons (65% of the total population) screened, 204 (1.36%) were CLIP-PCR positives. Among them, 188, 14, and 2 were infected with *Plasmodium vivax, Plasmodium falciparum*, and *P. vivax*/*P. falciparum* mix, respectively. The testing capacity was 538 persons/day, with a cost of US$0.92 /person. The proportion of submicroscopic infection was 64.7%. All positive individuals received treatment within 72 h after blood collection.

**Conclusion:**

Using CLIP-PCR in MSAT in low transmission settings can support the malaria elimination efforts in the China-Myanmar border region.

**Supplementary Information:**

The online version contains supplementary material available at 10.1186/s12936-023-04449-x.

## Background

Mass screening and treatment (MSAT) for malaria is defined as testing of an entire population in a wide geographical area followed by treating only positive individuals, regardless of whether they have malaria symptoms. Such an approach provides important information on the epidemiology of malaria, which can be useful for further disease containment efforts [1], and is more likely to be accepted by the target population than mass drug administration [2]. Moreover, mass prophylactic treatment with primaquine to control *Plasmodium vivax* outbreaks could be dangerous because of a high rate of glucose-6-phosphate dehydrogenase deficiency (up to14.8%) in the population of the target district [3]. Although recently the MSAT strategy has received renewed attention in the context of malaria elimination, the World Health Organization (WHO) did not recommend MSAT as an intervention strategy to interrupt malaria transmission, as there is a lack of a highly sensitive and high throughput diagnostic tool that is both cost effective and field-friendly to operate [2, 4].

To overcome this challenge, a high-throughput molecular assay on a 96-well plate platform called Capture and ligation probe-PCR (CLIP-PCR) has been developed [5], enabling direct malaria RNA detection without nucleic acid purification and reverse transcription, with a limit of detection of 0.01 malaria parasite/µL blood or around 0.33 parasite/µL if using 3 mm dried blood spot (DBS) [5, 8]. This meets the target detection limit recommended by the WHO (2 parasites/µL or lower) for PCR-based malaria screening in low-transmission settings [6], while making malaria screening easier and less expensive than microscopy and standard nested PCR [7, 8]. In this method, 18 S rRNA targets in blood samples are released through cell lysis and captured by a series of specific, tailed probes onto the wells of a 96-well capture plate through sandwich hybridization. After removal of extra probes and impurities, bound tailed probes are ligated to form single stranded templates for subsequent qPCR amplification and detection [5]. Recently, an improved version of CLIP-PCR, the multi-section CLIP-PCR (mCLIP-PCR), was developed enabling *Plasmodium* genus- and species-identification with a much-shortened assay time [9].

Kachin Special Region II (KSR2), a poor mountainous area located in northern Myanmar, shares a land border of 214.6 km with Yingjiang County of Yunnan Province, China. Since the regional conflict in KSR2 in 2012, a large number of internally displaced persons immigrated and resettled along the China-Myanmar border, posing a risk to the achievements of joint cross-border malaria prevention and control activities between China (Yunnan Province) and Myanmar [10–14]. In KSR2 of Myanmar, the incidence of malaria increased from 2.1% to 2012 to 5.1% in 2016 [15]. Besides, an outbreak of *P. vivax* was observed in Laiza City and the surrounding areas in 2016 [16], where the number of malaria cases increased sharply by 2.3 times from 940 in 2015 to 2080 in 2016 [16]. The number of malaria cases imported from Myanmar to Yingjiang County rapidly increased from 35 in 2012 [17] to 185 in 2016 [16], jeopardizing the goal of malaria elimination in Yingjiang County by 2020 [18–20].

In this study, the authors evaluated the feasibility of using CLIP-PCR in MSAT to determine the malaria prevalence in the Laiza district of Myanmar. Understanding the level of malaria prevalence in Laiza district can provide a basis for controlling the *P. vivax* outbreak and reducing the cross-border malaria importation into Yunnan, China.

## Methods

### Study areas and population

**Fig. 1:**
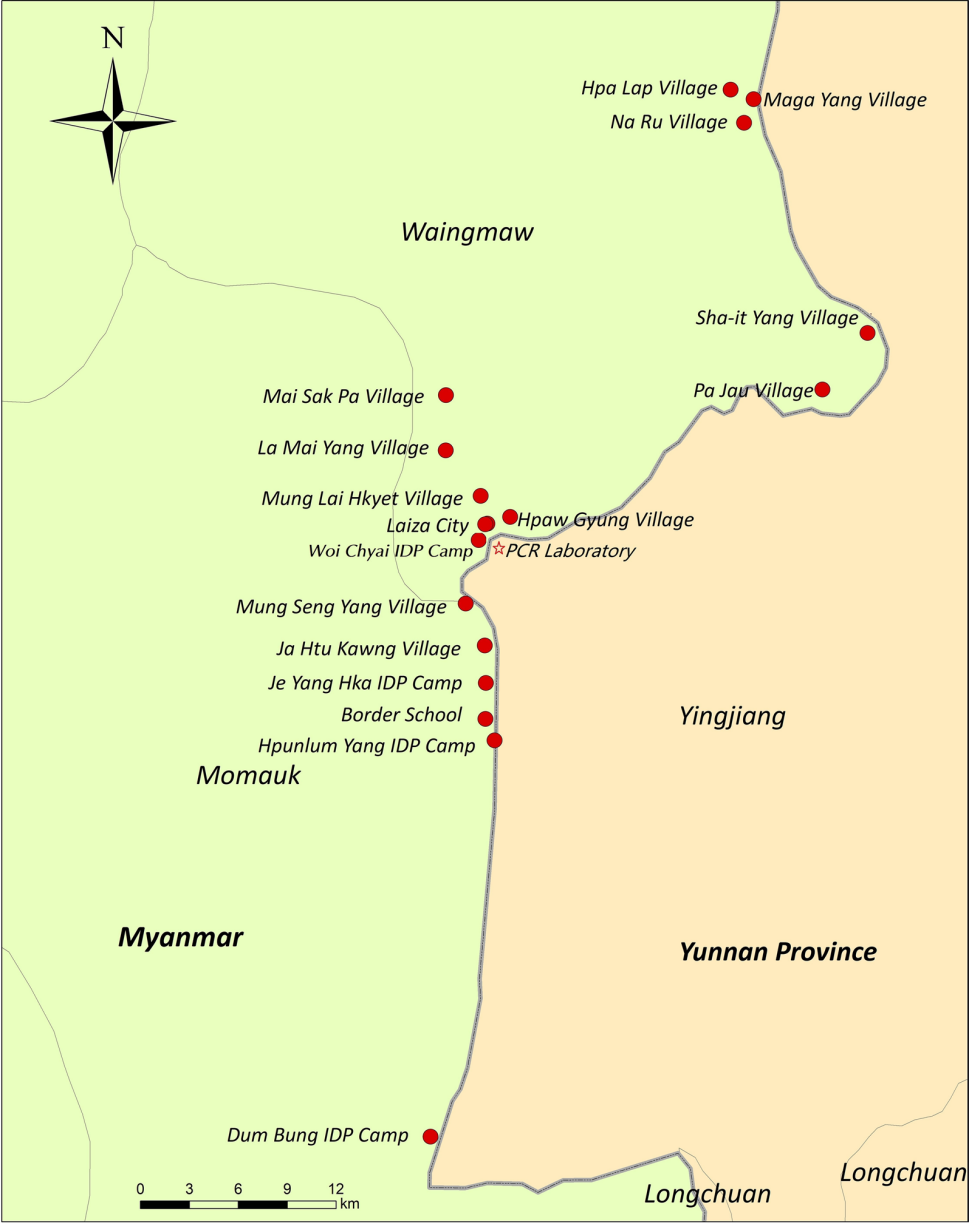
The study areas of MSAT for malaria

The study was conducted from April to May 2017. All Laiza district areas within 2.5 km from the border with China were covered, including urban and suburban regions of Laiza City, six surrounding villages (Hpaw Gyung, Ja Htu Kawng, Mai Sak Pa, La Mai Yang, Mung Lai Hkyet, and Mung Seng Yang), five distant villages (Hpa Lap, Maga Yang, Na Ru, Pa Jau, and Sha-It Yang), four internally displaced persons (IDP) camps (Dum Bung, Je Yang Hka, Hpum Lum Yang, and Woi Chyai), one school and two army camps. The total population included was 23,169 individuals residing in brick concrete bungalows in Laiza City, wooden tile houses in villages, and temporary sheds made of iron sheets and asbestos tiles in IDP camps. The study areas were depicted in Fig. 1.

### Main reagents and equipment

*Plasmodium falciparum* (Pf)/Pan-LDH rapid diagnostic test (RDT) was the product of Guangzhou Wondfo Biotechnology Co., Ltd., China (lot W05460602WC). CLIP-PCR 2.0 kits were purchased from Diacurate Technology Co., China (Daanrui@126.com), and multi-section CLIP-PCR (mCLIP-PCR) kits for *Plasmodium* genus and species identification were donated by Peking Union Medical College, Beijing. The related probe sequences are provided in Additional file 1: Table S1. An Omron non-contact infrared forehead thermometer (model MC-720) was the product of Kunshan Reying Photoelectric Co., Ltd. (China), a real-time fluorescence quantitative PCR (qPCR) instrument (model CG-5) was purchased from Shanghai Likang Biomedical Technology Holding Co., Ltd. (China), and a shaking incubator (VorTemp 56) was obtained from Labnet International (USA).

### Polymerase chain reaction (PCR) laboratory

**Fig. 2:**
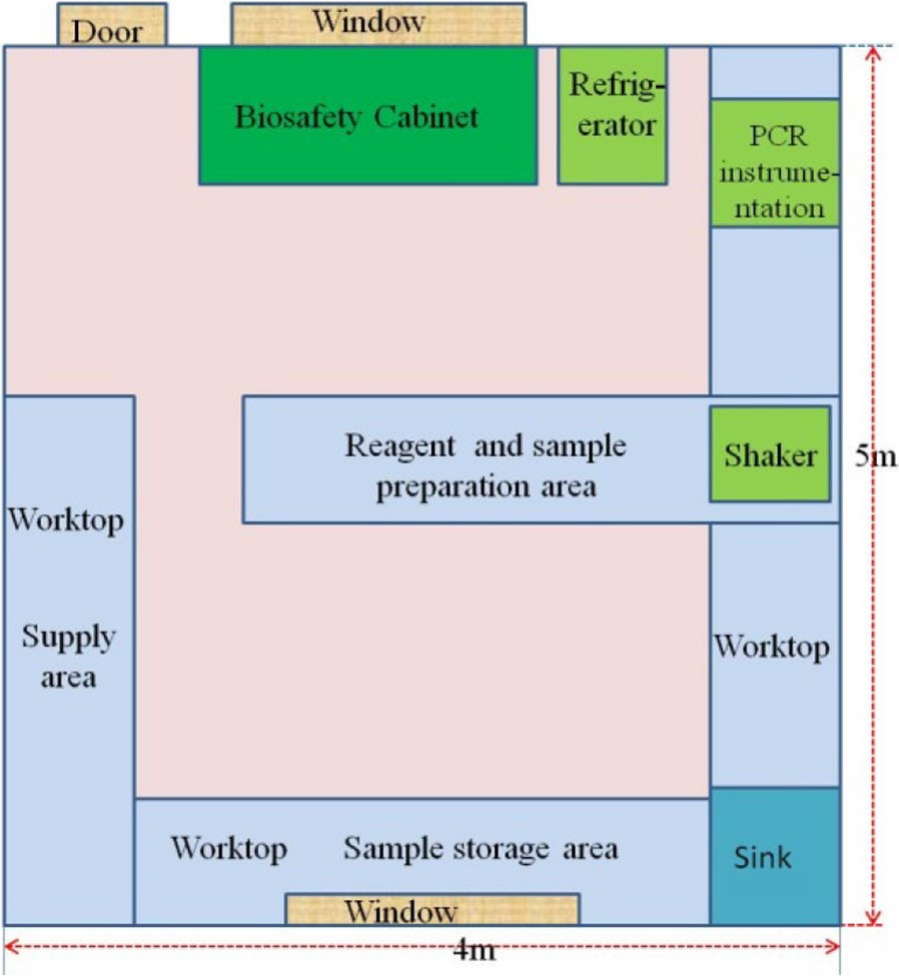
The PCR laboratory layout

A PCR laboratory was established in a single room of 20 m^2^ on the first floor of the outpatient department in the Nabang township health clinic (Yingjiang County, China). Worktops covered with ceramic tiles were positioned along the three walls and in the middle of the room. A two-person single-side vertical laminar flow biosafety cabinet was set near the front window at the entrance, and a standard refrigerator was placed to the right of the biosafety cabinet. The layout of the PCR laboratory is shown in Fig. 2.

### Blood sample collection

Sampling was performed by seven trained healthcare workers, who notified the target community one day before the procedure. On the sampling day (day 0), a short community meeting was held to read out the informed consent form and deliver information on malaria and the associated health issues. Individuals were invited to participate voluntarily and signed informed consent. After registration of the blood test form, the body temperature of each participant was measured with a non-contact infrared forehead thermometer and two blood spots were collected with a trifold Whatman 903 Specimen Collection card. The blood spots on the open trifold card were dried briefly in a ventilated room by hanging the card on a rope for 30–120 min. The cards were then folded and brought to the laboratory in a special storage box on the same day. If a participant had body temperature ≥ 37.8 °C or reported fever history within the past week, a rapid diagnostic test (RDT) was performed at the time of blood sample collection and a blood smear was prepared in case of a positive result.

#### ***Plasmodium*spp. screening by CLIP-PCR**

Four technicians of the testing group placed the blood spot samples collected by the sampling group on the laboratory worktop for overnight drying. The next day (day 1), two dried blood-containing discs 3 millimeters (mm) in diameter were punched from one blood spot of each participant, and screened for *Plasmodium* spp. using CLIP-PCR with a matrix pooling strategy [5, 7]. Briefly, samples were placed in a 10 × 10 matrix. One disc from each sample in a raw was pooled, and separately one disc from each sample in a column was pooled. To prevent potential cross-contamination, after each punch, the metal puncher head was dipped in 70% alcohol and flamed over an alcohol burner for 3–5 s, let cool and punch three times on a blank filter paper, before next use on a different blood spot. Pooled discs were placed into a 1.5 mL centrifuge tube and incubated with 170 µl (µL) of lysis solution for 30 min with shaking (1200 rpm) at 56 °C to lyse the cells. Then, 100 µL was transferred to each well of a 96-well capture plate and was added 1 µL of probe mix for capturing 18 S rRNA of genus *Plasmodium*. The solution was incubated with shaking (800 rpm) for 120 min at 56 °C to capture the target onto the plate. After plate-washing the bound detection probes on the target were ligated at 37 °C for 10 min to form a template for subsequent qPCR amplification in the same well with SYBR Green chemistry, using primers specific for the genus probes. For positive raw-pools and column-pools, their “intersection” samples were candidate positives and were further confirmed by assaying the individual samples without pooling, using CLIP-PCR for either *Plasmodium* spp. or *Plasmodium* genus and species (see below), while others were declared negative.

Throughout screening cultured *P. falciparum* 3D7 lysate was used as kit positive control and was placed randomly in the plate; several no-template controls were used as negative controls placed in wells next to the positive control. As an internal control to validate CLIP-PCR, blood smears of Day 0 RDT-positive participants were first confirmed by microscopy according to WHO recommendations [21, 22], the corresponding blood spots were included, in a random manner, in the group of samples collected on the same day for subsequent CLIP-PCR tests by four technicians blind to sample identity.

#### ***Plasmodium*genus and species determination by mCLIP-PCR**

The positives from areas where *P. falciparum* was reported in the preceding two years were analysed for genus and species determination by multi-section CLIP-PCR (mCLIP-PCR) [9]. Briefly, a dried blood disc 3 mm in diameter was placed into a 1.5 mL centrifuge tube and incubated with 120 µL of lysis solution for lysis. Afterwards 100 µL was transferred to a well of a 96-well capture plate and was added 1 µL of probe mix, containing both genus probe mix and species probe mix, for capturing *Plasmodium* genus-specific, *P. falciparum* species-specific and *P. vivax* species-specific sections of 18 S rRNA onto the plate. After plate-washing and a 50 µL probe-ligation reaction, the plate was heated for 1 min at 90 °C to release the ligated products, and 5 µL was transferred to a new PCR plate well for genus identification by qPCR performed in a total volume of 25 µL/well containing primers specific for the genus probes. Genus-positive samples were further assayed for species identification by transferring another 5 µL to a new well for qPCR, either with primers for the *P. falciparum* species-specific probes, or with primers for the *P. vivax* species-specific probes.

### RDT confirmation and treatment

On day 2, positive CLIP-PCR or mCLIP-PCR results were provided to a clinician who was responsible for the follow up of the participants. An additional RDT was performed on these positive participants to confirm some of the positives, and to see how many of these positives were below the detection limit of RDT. Regardless of the RDT results treatment was initiated to each positive participant according to WHO recommendations.

### Statistical analysis

The database was generated using Microsoft Excel 2010 and analyzed using descriptive statistics (SPSS Statistics version 20). The daily testing capacity was expressed as the number of CLIP-PCR-screened participants per day. The screening cost per person was determined as the actual purchase price of CLIP-PCR reagents divided by the number of screened participants. The CLIP-PCR-determined parasite prevalence rate (%) was calculated as the number of positive results divided by the total number of screened participants. In RDT confirmation of PCR results, the samples were regarded as microscopic when RDT results were positive and submicroscopic when RDT results were negative. The percentage of submicroscopic infections was calculated relative to the number of positive samples.

## Results

### Blood sample collection

A total of 15,038 participants were sampled from April 26 to May 23, 2017, with a population coverage rate of 64.91% (15,0381/23,169). There were 7260 men and 7778 women; the average age of the participants was 24.34 ± 17.66 years (range, 0.5–93 years). On Day 0, a total of 118 individuals were tested by the RDTs. Among them 75 had fever (body temperature ≥ 37.8 ℃) on site and 43 had fever history within one week, and 9 of the 118 were found RDT-positive. Among them, 8 were tested positive for *P. vivax* and 1 for *P. falciparum* by microscopy on Day 1.

#### Plasmodium spp. screening

A total of 204 positive samples were identified among the 15,038 blood samples using CLIP-PCR kits for 35 plates. The PCR-based parasite prevalence rate was 1.36% (204/15,038), the testing capacity was 537 samples/day (15,038/28), and the screening cost was about US$ 0.92 /person ($13,835/15,038). The 9 RDT-positive samples were confirmed by CLIP-PCR. The sensitivity and specificity of CLIP-PCR compared with microscopy were both 100%.

#### ***Plasmodium*genus and species determination**

Most of the surveyed areas in this study reported only *P. vivax* in the preceding two years and, therefore, individually-confirmed CLIP-PCR positive samples from these areas were considered *P. vivax* infections without further testing. The 53 CLIP-PCR positive samples from areas where *P. falciparum* was reported in the preceding two years were analysed for genus and species determination by multi-section CLIP-PCR (mCLIP-PCR). Among them, 53 were confirmed positive at the genus level by mCLIP-PCR with an agreement rate of 100% compared with CLIP-PCR. The species identification with mCLIP-PCR was 35 *P. vivax*, 14 *P. falciparum*, 2 *P. vivax*/*P. falciparum* mix and 2 negative. The 9 RDT-positive samples were identified at the species level by mCLIP-PCR with the same results as microscopy.

### RDT confirmation

On day 2, all of 204 CLIP-PCR positive participants were followed up with RDT confirmation. Among them, 132 of 204 were RDT negative and were regarded as submicroscopic or subpatent, giving rise to a submicroscopic infection rate of 64.71% (132/204). Among the 72 RDT positives, 9 were confirmed *P. falciparum* or mixed species positive and 63 were *P. vivax*- or other non-*P. falciparum* positive (Table 1). The results of species identification with mCLIP-PCR and RDT were shown in Table 2.

**Table 1:** The results of RDT confirmation for 204 samples screened positive by CLIP-PCR

RDT results	Number of cases
*P. vivax*- or other non-*P. falciparum-*positive	63
*P. falciparum*- or mixed species-positive	9
Negative	132
Total	204

**Table 2:** The results of species identification with mCLIP-PCR and RDT*

mCLIP-PCR determination		RDT confirmation		% RDT confirmed
*P. vivax*	35	*P. vivax*- or other non-*P. falciparum* species*-*positive	21	60%
*P. falciparum*	14	*P. falciparum*- or *P. falciparum/P. vivax*-positive	9	56%
*P. falciparum+P. vivax*	2			
Genus positive but both species negative	2	Negative	23	–
Total	53	Total	53	–

*Those CLIP-PCR positive samples from areas where both *P.vivax* and* P. falciparum* cases were reported in the preceding 2 years were subjected to species-identification by mCLIP-PCR and RDT

### Treatment and geographical distribution

**Fig. 3:**
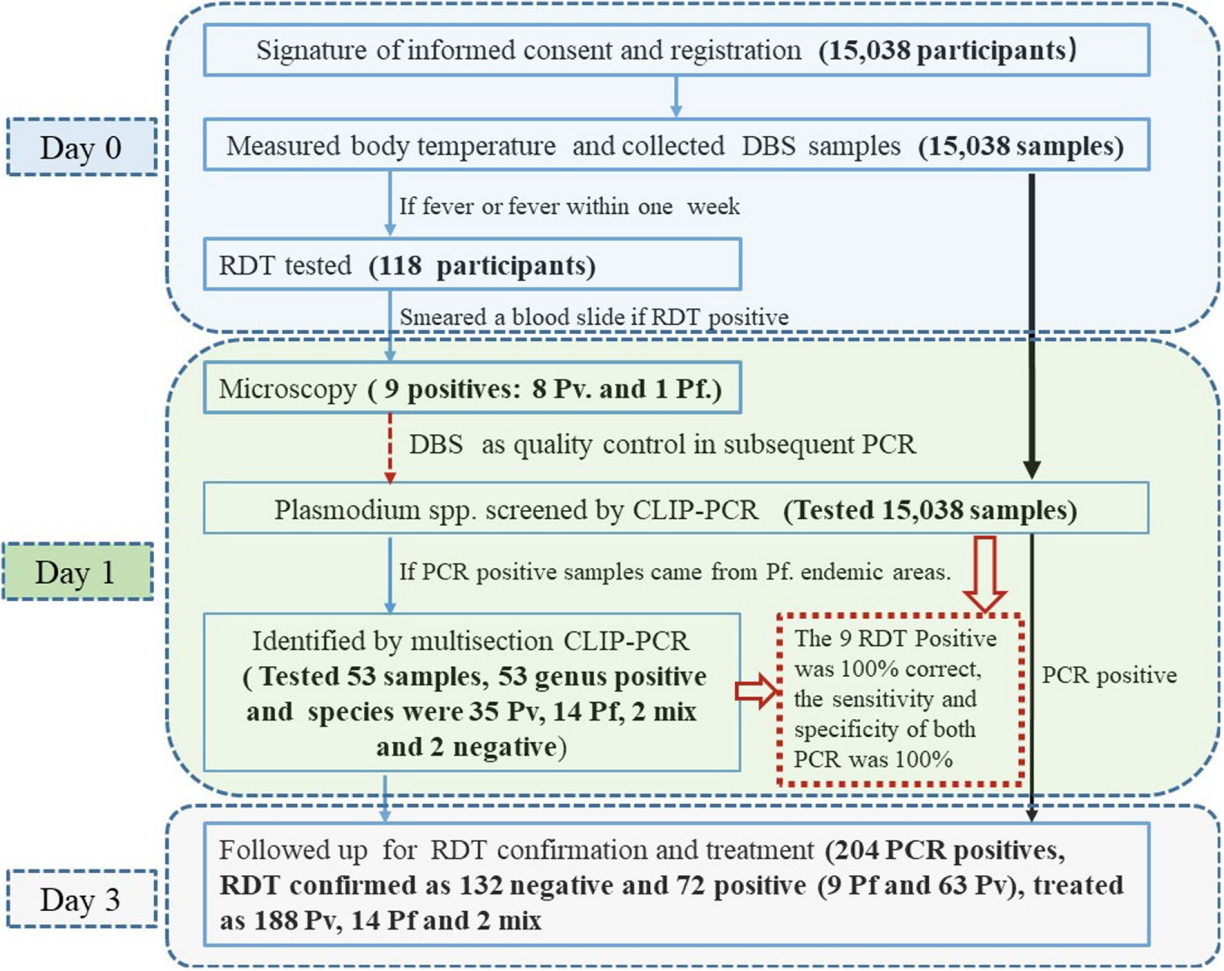
The flow chart of the screening. (1) The boldface in the brackets were the results. (2) DBS: Dried Blood Spots. (3) RDT: Rapid Diagnosis Test. (4) Pv *P. vivax.* (5) Pf *P. falciparum.*

The 204 patients screened positive with CLIP-PCR received treatment, among them, 188, 14, and 2 were treated as *P. vivax*, *P. falciparum*, and mixed *P. falciparum* + *P. vivax*, respectively. The proportion of *P. vivax*-infected participants was 92.2% and the ratio of *P. falciparum* to *P. vivax* infection was 0.09. The geographical distribution of malaria is summarized in Table 3. The MSAT screening workflow in this study is outlined in Fig. 3.

**Table 3:** Malaria geographical distribution in the study areas

Study area	Distance from Laiza City	No. of screened participants	No. of screened positive	The PCR-detected parasite prevalence rate (%)	Final species identification (No.)		
					P.v	P.f	Mix
Sha-it Yang Village	Far	89	17	19.10	4	11	2
Laiza City	–	2903	82	2.82	82	0	0
Je Yang Hka IDP Camp	Near	3589	74	2.06	74	0	0
Ja Htu Kawng Village	Near	108	2	1.85	1	1	0
Mai Sak Pa Village	Near	229	3	1.31	1	2	0
Mung Seng Yang Village	Near	396	4	1.01	4	0	0
Woi Chyai IDP Camp	Near	1114	7	0.63	7	0	0
Hpunlum Yang IDP Camp	Near	1981	11	0.56	11	0	0
Dum Bung IDP Camp	Near	227	1	0.44	1	0	0
Border School	Near	528	1	0.19	1	0	0
Army Camps	Near	1827	2	0.11	2	0	0
La Mai Yang Village	Far	34	0	0	0	0	0
Mung Lai Hkyet Village	Far	700	0	0	0	0	0
Hpaw Gyung Village	Far	144	0	0	0	0	0
Pa Jau Village	Far	398	0	0	0	0	0
Hpa Lap Village	Far	47	0	0	0	0	0
Maga Yang Village	Far	603	0	0	0	0	0
Na Ru Village	Far	121	0	0	0	0	0
Total		15,038	204	1.36	188	14	2

## Discussion

For decades since the invention of PCR, most molecular diagnostic innovations focused exclusively on amplification and detection to improve the sensitivity and specificity, but few focused on preanalytical process to reduce the complexity of molecular assays, which is the real impediment to their widespread applications. CLIP-PCR is conceptually similar to ELISA, with the capture probe and detection probe being analogous to the capture antibody and detection antibody. Subsequent PCR amplification is a signal amplification (rather than target amplification) analogous to the enzymatic cascade for signal detection in ELISA. Furthermore, the ligation step in CLIP-PCR affords two important benefits ELISA cannot have: (1) significantly better specificity and reduced background, as individual probes will not generate signal; and (2) ability to significantly avoid contamination, as it is only the ligated probes that are being detected. The reduced complexity of CLIP-PCR makes it possible to achieve high-throughput and sensitive molecular screening.

Contamination, high cost associated with tedious procedures, and low throughput are some of the challenges in PCR-based detection. Therefore, the WHO requires that a laboratory performing PCR analyses with diagnostic purposes be divided into at least three physically separated compartments for reagent preparation, sample preparation, and amplification and product detection [22, 23]. In this study, CLIP-PCR was successfully conducted in one room with only a biosafety cabinet, a refrigerator, a desktop shaking incubator (Shaker) and a qPCR machine (Fig. 2), suggesting that it is feasible to accomplish CLIP-PCR-based diagnostics regardless of laboratory space compartmentalization, provided that standard measures to prevent contamination are observed. For example, standard cares were taken to prevent cross-contamination at the sampling stage (e.g., by use of trifold sampling cards, and flaming of puncher head after each punch). In CLIP-PCR, most of the target RNA is captured to the bottom of the well, not in the solution, making it less prone to aerosol-related cross-contamination. Any unbound, cross-contaminating target RNA is unable to be a source of signal as, most importantly, what is amplified is not the target RNA but instead the ligated bound probes, which remain bound at the bottom. The amplification template does not form until after the ligation step ligated the bound probes right before amplification. These features make CLIP-PCR less prone to cross-contamination and suitable for operation in a multi-well plate format even at a room other than dedicated PCR clean-room. This study also demonstrated that a CLIP-PCR laboratory setup bypassing nucleic acid extraction would help solve the challenges of timely PCR diagnosis and prompt treatment [2, 24, 25], with a solution for MSAT for malaria: sampling on day 0, PCR screening on day 1, and treatment on day 2. In this study the CLIP-PCR testing capacity was 538 persons/day and the screening cost was only US$0.92 /person. Although it has been reported that CLIP-PCR detection can be conducted by pooling up to 36 samples/well [5] and that the cost per sample decreases even more with the increase in the number of pooled samples [7], the matrix pooling strategy for high-throughput screening requires concentration from the laboratory staff, and the possibility of a sample mishandling increases with the sample pooling number. Therefore, this study decided on a 10 × 10 matrix pooling strategy, with a double-checking mechanism intrinsic to the matrix pooling approach to minimize possible error (i.e. each positive sample should also have its raw-pool and column-pool both positive. If not, something is wrong). The screening capacity was limited to < 600 persons/day per qPCR machine, based on the sample collection speed and processing capacity of the shaker and PCR machine.

Compared with “ordinary” PCR with pooling [26, 27], which is another efficient and valid means for large scale malaria screening, CLIP-PCR with pooling has the following important advantages: (1) It does not involve nucleic acid extraction, therefore is more amenable to high throughput processing, and is much less concerned with sample contamination or nucleic acid degradation during- or post-extraction; It is also much less expensive than using automated DNA/RNA extraction devices. (2) The CLIP-PCR assay can test pooled DBS samples without losing sensitivity [5], as all the background RNAs from the negative samples are washed away without interfering the subsequent amplification. In contrast, in standard PCR with pooling, either the extraction or the amplification efficiency may be adversely affected in pooled samples, when interfering background nucleic acids in negative samples were added. (3) CLIP-PCR is less prone to cross contamination than ordinary PCR. CLIP-PCR does, however, require a qPCR machine, which can be too costly for laboratories in resource-limited countries. A similar strategy using LAMP [28] may reduce the overall setup cost.

The performance of CLIP-PCR, nested PCR, and quantitative reverse transcription-PCR (qRT-PCR) have been evaluated by Zhao et al. in testing of 1,005 individual samples collected from Laiza City and surrounding areas in 2015 [29]. The author claimed significantly lower sensitivity of CLIP-PCR than the other molecular methods [29], in contrast to similar field comparisons from the same reported area [7–9]. One important flaw in Zhao et al. [29] as a comparison study was that the CLIP-PCR was evaluated using only dried blood spot (DBS) samples with unknown quality, while nested PCR, qRT-PCR were all evaluated with fresh blood sample only [29]. No attempt was made to evaluate the nested PCR, qRT-PCR using the same DBS samples, while when CLIP-PCR was evaluated using standard blood samples the same high sensitivity of 0.01 parasites/µL as the other molecular methods was observed by Zhao et al. [29]. Therefore, the conclusion of Zhao et al. [29] remains questionable. In a recent screening of 4,580 asymptomatic DBS samples, a side-by-side comparison of CLIP-PCR genus assay with standard qPCR on a subset of 100 asymptomatic DBS samples showed 100% agreement between the two methods, with high correlation (R^2^ = 0.81) between Cq values of the two methods [9].

As both CLIP-PCR and mCLIP-PCR were originally validated with standard qPCR using dried blood spots [5, 9], and as most standard PCR setups require high-level laboratory conditions and are not suitable for high-throughput screening [24], this study no longer verified CLIP-PCR results using other PCR protocols in the field laboratory. Instead, quality control with no-template controls, kit positive controls (cultured *P. falciparum* 3D7 lysates), as well as 9 RDT-positive samples identified on day 0 in the same population, was used. The RDT positives included 8 confirmed positive *P. vivax* and 1 confirmed positive *P. falciparum* by microscopy on day 1. Blind screening with CLIP-PCR indicated 100% positivity, and mCLIP-PCR identified the expected species.

As shown in Table 3, according to PCR results malaria prevalence was the highest in Sha-it Yang Village (19.10%), which can be attributed to its location. Sha-it Yang Village is far from Laiza City (Fig. 1) in a remote valley close to the forest, where an ethnic army camp was set up, indicating a high risk of malaria transmission in conditions where medical service was not easily accessible [30]. The study findings also showed that malaria was mainly detected in Laiza City and nearby areas, with the highest prevalence in the city, whereas no cases were detected in distant areas. One reason for such distribution of cases could be that Laiza City is situated in a hot, low-altitude, densely populated area, where malaria transmission is perennial with a seasonal peak, whereas in the distant high-altitude areas (except for Sha-it Yang Village), the transmission is interrupted in the cold season [16]. Another reason is that this study started at the end of April 2017 – the early stage of the malaria epidemic peak in Laiza City, which experienced the *P. vivax* outbreak in 2016 [16]; thus, there could be a large number of asymptomatic carriers and newly infected individuals in Laiza City during the study period. This study showed that the total PCR-based parasite prevalence rate in the analyzed population was 1.36% and that the *P. falciparum*/*P. vivax* ratio was 0.09, which are indicative of a very low transmission area according to the WHO categorization [31]. The proportion of subpatent infection was 64.7%, which was similar to the 67% rate of submicroscopic *P. vivax* infection reported by Moreira et al. [32] and Cheng et al. [33], indicating a very large potentially infectious parasite reservoir in the study area.

This study has some limitations. Only 64.9% of the target population was covered by the screening. Although the sample collection team informed the target community one day in advance, some residents remained unwilling to volunteer, whereas others could not come in time, especially those who often work in the forest and are therefore at a high risk. This may have caused sampling bias. These results indicate that MSAT needs efficient logistics support. Furthermore, the PCR laboratory was established on the Chinese side of the border because of safety concerns, accommodation of researchers, and overall better conditions than in the Myanmar area under study. Although the laboratory was only 200 m away from some of the collection sites, it was not located in the field per se. Lastly, the rate of submicroscopic infection was calculated based on RDT results not on microscopy analysis, the actual number may be smaller.

## Conclusion

A strategy to include CLIP-PCR in MSAT for low transmission settings has the advantages of improved diagnostic sensitivity, high throughput, overall cost effectiveness, and timeliness of diagnosis and treatment. All these factors can be potential improvements to conducting MSAT in low transmission areas to support malaria elimination efforts in the GMS region.

## Supplementary Information

**Additional file 1: Table S1.** Probe sequences.

## Data Availability

The datasets used and/or analysed during the current study are available from the corresponding author on reasonable request.

## Abbreviations

CLIP-PCR: Capture and ligation probe PCR
KSR 2: Kachin special region II
mCLIP-PCR: Multi-section capture and ligation probe PCR
MSAT: Mass screening and treatment
PCR: Polymerase chain reaction
qPCR: Real-time fluorescence quantitative PCR
RDT: Rapid diagnosis test
